# Evaluation of Immune Responses Induced by *GRA7* and *ROP2* Genes by DNA Vaccine Cocktails Against Acute Toxoplasmosis in BALB/c Mice

**Published:** 2018

**Authors:** Hossein Vazini, Fatemeh Ghafarifar, Zohreh Sharifi, Abdolhosein Dalimi

**Affiliations:** 1. Department of Nursing, Faculty of Basic Sciences, Hamedan Branch, Islamic Azad University, Hamedan, Iran; 2. Department of Parasitology, Faculty of Medical Sciences, Tarbiat Modares University, Tehran, Iran; 3. Blood Transfusion Research Center, High Institute for Research and Education in Transfusion Medicine, Tehran, Iran

**Keywords:** BALB/c, DNA vaccine, *GRA7*, *ROP2*, *Toxoplasma gondii*

## Abstract

**Background:**

The severe damages of toxoplasmosis clearly indicate the need for the development of a more effective vaccine. Immunization with plasmid DNA is a promising vaccination technique. Therefore, *GRA7* plasmid was prepared to be used as a vaccine. The purpose of this study was evaluation of immunization with cocktail DNA vaccine including plasmids encoding *Toxoplasma gondii ROP2* and *GRA7* in BALB/c mice.

**Methods:**

In this study, 733 *bp* of *GRA7* gene was cloned in pCDNA3.1 plasmid as an expression vector. The plasmids containing *GRA7* and *ROP2* genes were administered *via IM* according to immunized mice three times with a 3 week interval. For lymphocyte proliferation and cytokine assay, splenocytes of immunized mice were cultured for proliferation and cytokine assay. The other mice in each group were inoculated by the parasite and mortality of the mice was evaluated on a daily basis.

**Results:**

The cytokine assay results and lymphocyte proliferation response in cocktail DNA vaccines showed that IFN-γ levels were significantly higher than controls (p<0.05), whereas IL-4 expression level in BALB/c mice immunized with cocktail was lower than that in control groups and these results are confirmed by MTT test. Predominance of the levels of IgG2a over IgG1 was observed in sera of the immunized mice. Furthermore, IgG2a values in cocktail DNA vaccines pcGRA7 were significantly higher than control group (p<0.01). In contrast, IgG1 antibodies were similar between the two groups (p>0.5). So, survival time in the immune groups was significantly prolonged in comparison to control ones (p<0.01).

**Conclusion:**

The immunized mice by DNA vaccine produce higher titration of IFNγ, indicated with Th1 response which is confirmed by high level of IgG2a. These data demonstrate that the cocktail *GRA7/ROP2* is a potential vaccine candidate against toxoplasmosis.

## Introduction

Infection with the intracellular parasite *Toxoplasma gondii (T. gondii)* is responsible for toxoplasmosis in humans and other warm-blooded animals. The zoonotic parasite *T. gondii* is an obligate intracellular pathogen able to infect all warm-blooded animals with high prevalence ^1,2^ . During infection, the parasite disseminates through the body and remains present under the form of tissue cysts, which are kept under control, but are not eliminated by the host’s cellular immune response ^1,3^ . In healthy animals and humans, most Toxoplasma infections occur unnoticed. However, in pregnant women, a primary infection during pregnancy may lead to infection of the fetus and congenital toxoplasmosis ^4^ . The consumption of raw or undercooked meat products from *T. gondii* infected animals is regarded as the most important source of transmission to pregnant woman, next to *T. gondii* oocysts shed in cat feces ^3^ . Infected meat has been shown to be a considerable risk for human infection ^5,6^ . Vaccination studies in mice have focused on the selection of protective antigens and the most promising experimental vaccines now combine proteins from micronemes, dense granules, and rhoptry organelles that are secreted by the parasite during active invasion of the host cell ^7^ . Immunization of mice with these cocktail DNA vaccines can offer more than 80% reduction in tissue cyst formation ^7,8^ , and the protection elicited by these vaccines is correlated to antigen-specific production of the cytokine IFN-γ ^9,10^ . The aim of this study was determination of DNA vaccine with complete genes of dense granule proteins *GRA7* and *ROP2* as DNA vaccine in BALB/c mice model. *GRA7* and *ROP2* could express in two important stages of Toxoplasma life cycle, tachyzoite and bradyzoite.

## Materials and Methods

### Ethics statement and animals

This project was approved by Ethical Committee of School of Medical Sciences of the Tarbiat Modares University [adopted from the Declaration of Helsinki (1975] and the Society for Neuroscience Animal Care and Use Guidelines (1998), approved implementation by the Medical Ethics Committee (April 2011). Female BALB/c mice aged 6 weeks were purchased from the Animal Center of Iran’s Razi Serum and Vaccine Production Research Institute and maintained under specific-pathogen-free conditions. All experimental protocols were in accordance with the guidelines for the care and use of laboratory animals of Tarbiat Modares University.

### Parasites, antigens and antisera

*T. gondii* tachyzoites (RH strain) were harvested from the peritoneal cavity of infected BALB/c mice 4 days after intraperitoneal (*IP*) injection. Tachyzoites of *T. gondii* were separated from infected mice, then purified from macrophages by filtration. *T. gondii* Lysate Antigen (TLA) was prepared by freezing and thawing method. The concentration of antigen was measured by Bradford method. The prepared antigen was frozen at −20°*C* until use.

### Preparation of recombinant plasmid

The primers were designed and synthesized according to the published DNA sequences from the GenBank database as listed in table 1. All target DNA fragments were amplified by Polymerase Chain Reaction (PCR) and cloned initially into the cloning plasmid pTOPO (TaKaRa, China), verified by sequencing and released by digestion with appropriate restriction enzymes, then subcloned into corresponding restriction enzyme recognition sites of the expression plasmid pcDNA3.1 (Invitrogen, USA). The 733 *bp* fragment of *GRA7* (GenBank No.Y13863, sequence positions 135–784) was amplified by PCR from the genomic DNA of *T. gondii* RH strain, and cloned into the pTOPO and subcloned into pcDNA3.1 *via* BamH I, EcoR I and Hind III and EcoR I restriction enzyme digestion, respectively, then generated the pcDNA3-GRA7 (pcGRA7). The expression plasmid pcDNA3-ROP2 (pcROP2), encoding the full-length *ROP2* (1686 *bp*, 64 *kDa*) antigen, was kindly provided by Dr. Hoseinian Khosroshahi (Tarbiat Modares University, Iran) ^11^ . All nucleotide sequences introduced into vectors were verified by DNA sequencing. The recombinant plasmids were transformed into *Escherichia coli (E. coli)*, strain TOP10. Mass replication of the bacterium was extracted from the bacteria using Endotoxin free plasmid extraction kit (Qiagen, Germany).

**Table 1.** Total IgG antibodies detected by ELISA in pooled sera of immunized mice (values are expressed as mean±SD)

**Number of group**	**Immunization regimen**	**OD of total IgG M±SD**	**Sig. differences with groups (p<0.05)**
**1**	PBS	0.31±0.05	(3,4,5,6)^*^
**2**	pcDNA3	0.52±0.15	(3,4,5,6)^*^
**3**	pcGRA7	1.16±0.23	(1,2,5,6)^*^
**4**	pcROP2	0.98±0.12	(1,2,5,6)^*^
**5**	pcROP2+pcGRA7	1.44±0.31	(1,2,3,4)^*^
**6**	TLA	1.68±0.38	(1,2,3,4)^*^

* According to the Mann–Whitney method, there was statistically significant difference between marked groups (p<0.05)

**Primers of *GRA7* (733 *bp*):**
Forward contain 26 *bp* with BamH1 restriction enzyme site:
  Forward: 5′ GCC-GGA-TCC-ATT-TCC-AAA-ATG-GCC-CG 3′
Revers 25 *bp* with EcoR1 restriction enzyme site:
  Reverse: 5′GAA-TTC-GCC-CCC-ATA-TCC-TAC-TGG-C 3′

**Primers of *ROP2* (1686 *bp*):**
Forward 32 *bp* with Hind III restriction enzyme site:
  Forward: 5′ ATT AAG CTT ATG GAA AAC TGT GCG TCG GTC AG-3
Revers 29 *bp* with EcoRI restriction enzyme site:
  Revers: ATT GAA TTC TCA TGC CGG TTC TCC ATC AG-3

### In vitro expression of construct pcGRA7 in mammalian cells

Chinese Hamster Ovary cells (CHO-K1) were transfected with pcGRA7 or a control plasmid pcDNA3.1 (Invitrogen, CA, USA), using fuGENE 6 transfection reagent (Roche, Germany). Cells were cultured in Dulbecco’s Modified Eagle Medium (DMEM) (Gibco, Invitrogen, USA) with 10% FCS and 100 *μg/ml* of streptomycin/penicillin at 37°*C* in 5% CO _2_ . Freshly grown CHO-K1 cells were seeded at 3×10 ^6^ cells/well in 500 *μl* of growth medium the day prior to transfection. Cells were transfected with plasmids using fuGENE 6 transfection reagent according to manufacturer’s instruction. Cells were grown for a further 24 and 48 *hr* before the cell pellets were collected and analyzed for transgene expression. For immunoblots with transfected cells, SDS-PAGE and immunoblotting were performed. The separated proteins were probed with anti-T. gondii human serum. Bound antibodies were detected with peroxidase-labelled goat antihuman IgG (DAKO, Denmark). Peroxidase activity was revealed as 3, 3′-Diaminobenzidine tetrahydrochloride substrate (Sigma).

### Immunization and infection of mouse

Six groups of mice (11 per group) were injected intramuscularly with 100 *μg* of plasmid DNA suspended in 100 *μl* sterile PBS, 50 *μl* in each thigh skeletal muscle, whereas negative control mice received PBS alone and positive control mice were immunized with TLA. Group I was injected with PBS as control, group II with empty pcDNA3 vector also as control, group III with pcGRA7 (50 *μg* of each purified pcDNA3-GRA7 and pcDNA3 plasmids injected together), group IV with pcROP2 (50 *μg* of each purified pcDNA3-ROP2 and pcDNA3 plasmids injected together), group V with pcGRA7+pcROP2 (50 *μg* of each purified pcDNA3-GRA7 and pcDNA3-ROP2 plasmids injected together). Group VI was injected with 50 *μg* of Toxoplasma Lysate Antigen (TLA) obtained from *T. gondii* RH strain mixed with adjuvant (1:1) and the first immunization was done with complete Freunds, the 2 ^nd^ and 3 ^rd^ immunization with incomplete Freunds with a 3 week interval. Mice were immunized using the same protocol on days 0, 21 and 42 and were bled by orbital plexus puncture on day 70 (four weeks after last vaccination). On day 70, six immunized mice per group were *IP* challenged with 5×10 ^4^ tachyzoites of virulent RH *T. gondii*.

### Lymphocyte proliferation assay by MTT [3-(4,5-methylthiazol-2-yl)-2,5-diphenyltetrazolium bromide]

Four weeks after the last booster immunization, spleens were surgically removed from euthanized mice (5 mice per group). In 96-well microtitre plates, 5×10 ^5^ per well were cultured in RPMI 1640 (Gibco) and 10% FCS (Gibco) and allowed to multiply for 72 *hr* in the medium alone (control group), in the presence of 40 *μg/ml* of Toxoplasma Lysate Antigen (TLA) and incubated at 37°*C* and 5% CO _2_ . After these times, 20 *μl* of tetrazolium (Roche, Germany) (5 *mg/ml*) was added to each well and incubated at 37°*C* for 4 *hr*, then centrifuged in 1000×*g* for 10 *min* and the supernatant was discarded and 100 *μl* of DMSO was added to each well and resuspended. The OD was read by ELISA reader at 540 *nm*.

### Antibody assay

The *T. gondii*-specific antibodies were analyzed by ELISA as previously described ^12^ . Briefly, microtiter plates coated with 10 *μg/ml* solution of total *T. gondii* antigens were used to capture antibodies in the mouse sera (sera were diluted 1/100 in 5% dried skimmed milk) and incubated for 2 *hr* at 37°*C*., which were then detected with horse radish peroxidase-conjugated goat anti-mouse IgG, IgG1, or IgG2a (DAKO, Denmark) as secondary antibodies for isotype analysis. Peroxidase activity was revealed by adding 50 *μl* per well of a solution containing 12.5% H _2_ O _2_ , 0.1 *M* citrate phosphate pH=4 and 10 *mg/ml* of TMB. The reaction was stopped by the addition of 1 *M* H _2_ SO _4_ and the absorbance at 490 *nm* was measured with ELISA reader (BiotekELx800, USA). For each serum sample, the assay was done in triplicates and average values were calculated and the data were statistically analyzed using SPSS software.

### Cytokine assays

Detection of cytokines was measured from 5 mice per group on the 4 ^th^ week after the final immunization as well as stimulation with TLA and viable splenocytes were dispensed into 96-well plates at 5×10 ^5^ cells/well. The culture was incubated at 37°*C* with 95% relative humidity and in the presence of 5% CO _2_ . Splenocytes were harvested and measured for interleukin-4 (IL-4) activities at 24 *hr* and for IFN-γ activity at 96 *hr* using a commercially available ELISA Kit (R&D Systems, USA).

### Statistical analysis

The survival time was analyzed and compared between the immunized and control groups using the Kaplan-Meier method. The difference in the level of cytokine and antibody production was determined by one-way ANOVA. All statistical analyses were performed using the SPSS software. Two-sided p<0.05 were considered to indicate statistical significance.

## Results

### Recombinant plasmid and expression of T. gondii GRA7 proteins in vitro in CHO cells

**Figure 1.**
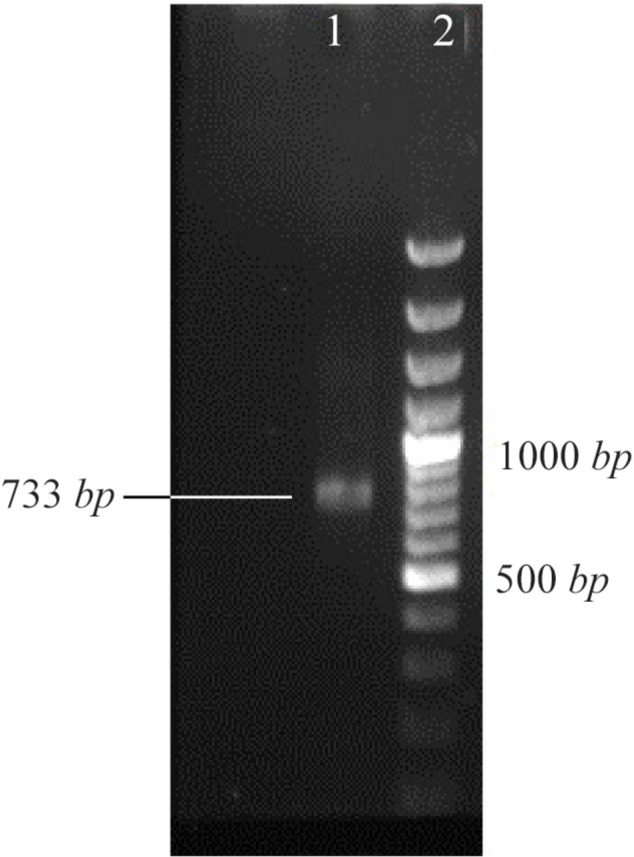
The PCR product of *GRA7* with 733 *bp* band (line 1) in comparison with 100 *bp* DNA ladder (line 2).

**Figure 2.**
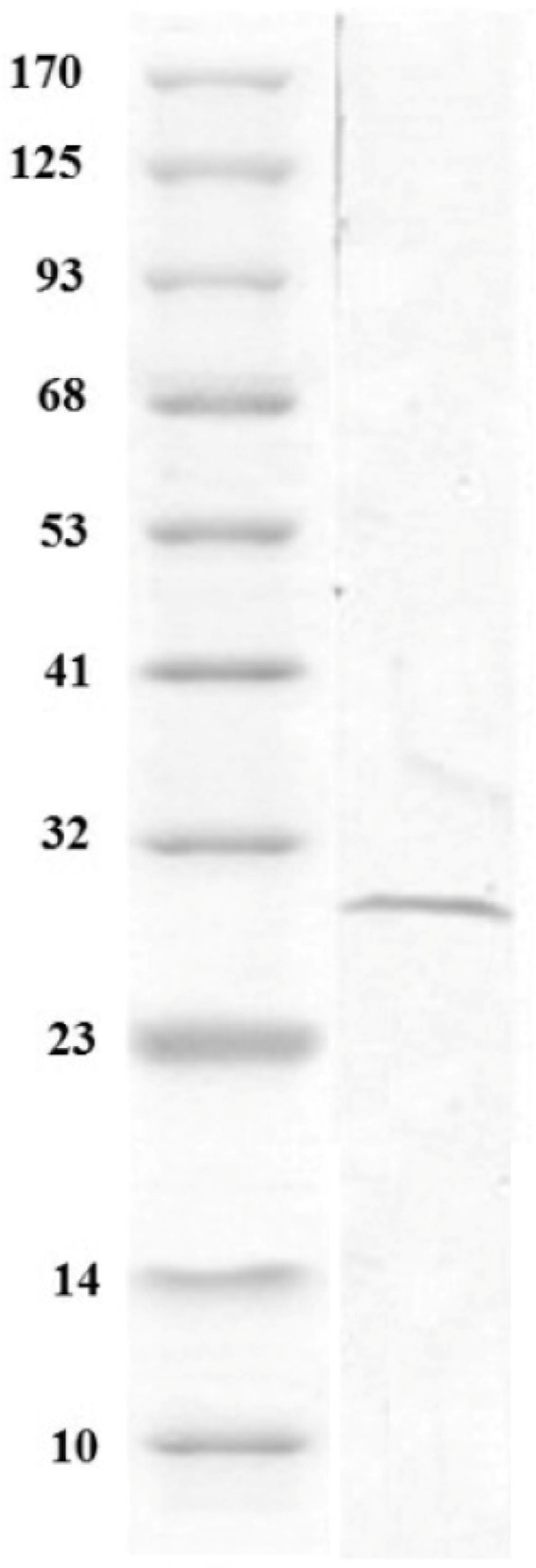
Immunoblotting of CHO cells with transfected inserted plasmids with *GRA7* genes of *T. gondii* showed protein with 29 *kD* molecular weight.

The results of constructions of pC-ROP2 have been reported previously ^13^ . In this study, a 732 *bp* of *GRA7* was cloned in pCDNA3.1 (Figure 1) and expression of protein *in vitro* was investigated in CHO-K1 cells. Cells were transfected with pcGRA7 or empty pcDNA3.1 for 24 *hr*. The protein extracts were then analyzed by SDS-PAGE and finally with western blotting (Figure 2). Immunoblotting of CHO-K1 cells transfected with inserted plasmids showed that *T. gondii* recombinant protein *GRA7* was expressed *in vitro* from these plasmid vectors. The production of *GRA7* was confirmed by immunoblot using polyclonal anti-T. gondii human antibodies.

Specific immune reactive proteins of approximately 29 *kDa* respectively were detected in cells transfected with pcGRA7 which were not present in cells transfected with the control plasmid vector pcDNA3.1. These results indicate that recombinant *GRA7* protein was successfully produced and secreted by mammalian cells.

### Evaluation of the survival rate in the immunized animals

**Figure 3.**
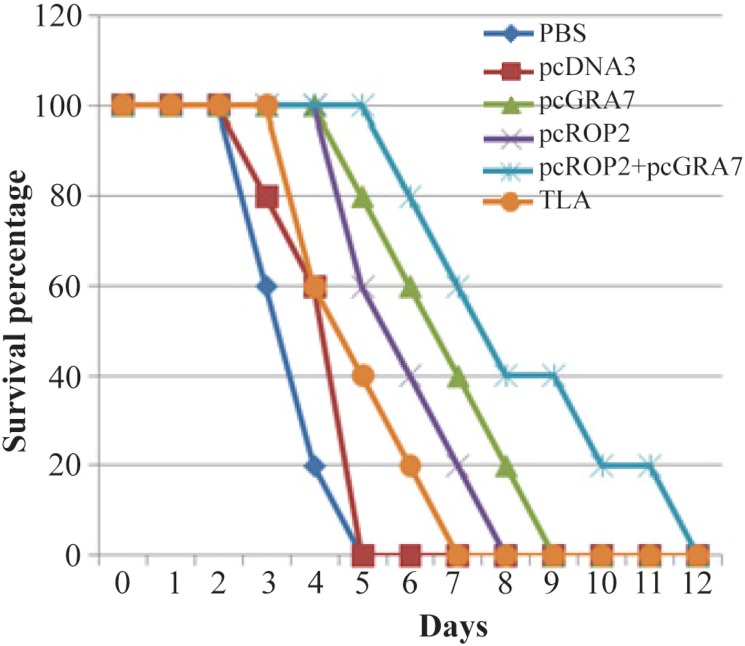
Survival curves of immunized BALB/c mice after lethal challenge with 5×10 ^4^ tachyzoite forms of RH *T. gondii* strain, 4 weeks after the last immunization. Each group has six mice.

To evaluate whether this vaccination protocol could elicit protection against *T. gondii*, immunized mice were challenged with tachyzoites of the virulent RH strain. Survival rates in those different groups of mice are shown in figure 3. Significantly higher survival rates were seen in pcGRA7+pcROP2 vaccinated mice compared with PBS, TLA, empty plasmid and singlegene plasmid pcDNA3-GRA7 and pcDNA3-ROP2 vaccinated mice. Mice immunized with PBS or pcDNA3 died within 3–4 days and immunized mice with TLA died within 4–7 days (Figure 3). Although all the experimental mice died, the survival time of the mice that immunized with pcGRA7+pcROP2 was markedly longer than those immunized with control groups and pc*GRA7* or *ROP2* groups (p<0.05).

### Humoral immune response induced by DNA vaccination

**Figure 4.**
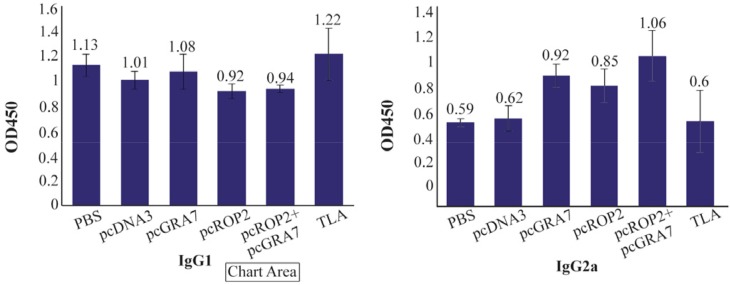
Results for level of immunoglubin G subtypes evaluation, IgG1 and IgG2a subtypes in mice serum samples of different experimental groups assayed by ELISA.

To investigate whether mice immunized with recombinant plasmids induced humoral immune response, sera collected from mice were tested by ELISA. The levels of total IgG antibodies in pooled sera of immunized mice (on days 42, 63 and 70) are shown in table 1. As shown in table 1, significant high levels of IgG antibodies were detected in the sera of mice immunized with pcGRA7+pcROP2, pcGRA7 or pcROP2 (p<0.05 versus control groups). There was significant antibody response as compared to the control group with pcDNA3 or PBS (p<0.05) (Table 1). Results also showed that pcGRA7+pcROP2 elicited IgG antibody values increased with successive immunizations as compared to single-gene plasmid pcGRA7 or pcROP2 (p<0.05). There was no significant difference in IgG responses between the groups immunized with pcGRA7 and pc*ROP2* (p>0.05). Meanwhile, mice immunized with pcGRA7+pcROP2 elicited higher levels of IgG2a as compared to pcGRA7 or pcROP2 (p<0.05), but there was no significant difference in IgG2a levels between the groups immunized with pcGRA7 and pcROP2 (p>0.05) (Figure 4).

### Cellular immune response induced by DNA vaccination

To investigate the possibility of cellular immune response in the immunized mice, the level of lymphocyte proliferation and cytokines in the spleen cell suspensions was evaluated 4 weeks after the final immunization (on day 56) as well as after stimulation with TLA. The pattern of MTT in immunized and control groups is shown in table 2. The lymphocytes proliferation assay results as indicated by MTT in all immunized groups in comparison with PBS and pcDNA3 groups were significantly higher (p<0.05) (Table 2). The differences between pcGRA7+pcROP2 group with separates pcGRA7 and pcROP2 groups were significant (p<0.05), but there was not any significant difference between pcGRA7 and pcROP2 groups (p>0.05).

**Table 2.** The mean±SD of OD (540 *nm*) detected by MTT in lymphocyte proliferation assay of vaccinated and control mice stimulated with TLA for 72 *hr*

**PBS (mean**±**S.D.)**	**pcDNA3 (mean**±**SD)**	**pcGRA7 (mean**±**SD)**	**pcROP2 (mean**±**SD)**	**pcGRA7+ROP2 (mean**±**SD)**	**TLA (mean**±**SD)**
0.41±0.14	0.385±0.11	0.93±0.22^*^	1.14±0.18^*^	1.87±0.37^*^	1.39±0.20^*^

* According to the Mann–Whitney method, there was statistically significant difference with control groups (p<0.05)

The levels of IFN-γ and IL-4 produced in splenocytes from immunized mice stimulated with TLA are shown in table 3. Significant high level of IFN-γ was observed in spleen cell cultures from mice immunized with pcGRA7+pcROP2 compared with mice immunized with PBS, empty plasmid and single-gene plasmids (p<0.05). On the other hand, low levels of IL-4 were observed in supernatants from spleen cells of pcGRA7+pcROP2 vaccinated mice compared with PBS, pcDNA3, pcGRA7, and pcROP2 groups (p<0.05).

**Table 3.** Cytokines detected by ELISA in splenocyte culture from immunized mice after stimulation with tachyzoite Toxoplasma Lysate Antigens (TLA)

**Number of group**	**Immunization regimen**	**(IFN-γ, *pg/ml*) Mean±SD**	**Sig. differences with groups (p<0.05)**	**(IL-4 *pg/ml*) Mean±SD**	**Sig. differences with groups (p<0.05)**
**1**	PBS	68.19±11.50	(3,4,5,6) ^*^	18.95±3.19	-
**2**	pcDNA3	91.35±18.34	(3,4,5,6) ^*^	12.23±4.07	-
**3**	pcGRA7	287.13±22.13	(1,2) ^*^	12.37±2.97	-
**4**	pcROP2	280.05±20.17	(1,2) ^*^	14.74±2.18	-
**5**	pcROP2+ pcGRA7	450.66±23.55	(1,2) ^*^	9.04±2.39	-
**6**	TLA	302.18±40.78	(1,2) ^*^	44.91±8.75	(1,2,3,4,5) ^*^

* According to the Mann–Whitney method, there was statistically significant difference between marked groups (p<0.05)

These findings confirmed the results obtained with the anti-T. gondii IgG subclass, indicating that the cellular immune response was also oriented to a Th1 profile in immunized mice.

## Discussion

The present study demonstrated that immunization with an enhanced pcGRA7+pcROP cocktail DNA vaccine is able to elicit a strong humoral and Type 1 cellular immune response characterized by the production of IFN-γ, against *T. gondii* infection in mice. It is well known that infection which elicited cellular immune responses are correlated to protection against *T. gondii* in mice and humans ^14–16^. After two immunizations with an enhanced cocktail DNA vaccine formulation, all vaccinated mice seroconverted against *GRA7*, *ROP2*, and this response increased considerably after last immunization. It was demonstrated that administration of a cocktail DNA vaccine is able to elicit humoral and cellular immune responses against *T. gondii* in mice.

The high incidence and severe or lethal damages of toxoplasmosis clearly indicate the need for the development of a more effective vaccine ^17^. In fact, protective immunity against this parasite is based mainly on CD8+T cells and is interfered by interferon-gamma-(IFN-γ) production ^10^. Also, recent studies have shown that DNA-based gene vaccination enables the production of the native form of a given antigen, priming both cellular and humoral specific immune responses ^18–21^.

In conclusion, our results show that the introduction of two functional genes encoding complete *GRA7* and *ROP2* as DNA vaccine cocktail elicits stronger Th1 type cellular immune response than single gene vaccines. Therefore, this immunization regime may be an important approach to access a multi-component vaccine against *T. gondii*, particularly with respect to generating an efficient long-lasting protective immune response. The results presented here provide a basis for further research towards the use of multicomponent DNA vaccines combined with cytokine plasmid and adjuvant in protection studies against toxoplasmosis.

The results of this study showed that DNA vaccine contain *GRA7* and *ROP2* could stimulate the cellular and humoral immunity. The antibodies in vaccinated mice were specific and the main subtype was the IgG2a whereas in TLA group, total IgG (Table 1) and IgG1 (Figure 4) were higher than other groups. The results indicated that TLA could stimulate the humoral immune response more than the DNA vaccine whereas DNA vaccine could stimulate the cellular immune response more than TLA. The results in table 2 showed that the lymphocytes of group V, which immunized with a cocktail of pcGRA7+pcROP2, proliferated more than TLA group. This result also confirmed that cocktail of DNA vaccine could stimulate the cellular immune response more than the TLA. In toxoplasmosis, both immune responses, the humoral and cellular, are important and for efficient protection, the stimulation of both is a need. On the other hand, the protection in toxoplasmosis is related to two tachyzoite and bradyzoite forms. In this study, the two antigens expressed in both tachyzoite and bradyzoite forms were selected. DNA vaccine with *GRA7* and *ROP2* genes could reduce the mortality of infected mice too. According to our results, it is suggested that these two genes could be good candidates for vaccines against toxoplasmosis caused by *T. gondii* and can be enhanced with other Toxoplasma genes or adjuvants. In our previous studies, three genes containing GRA5, SAG1, and *ROP2* could produce higher levels of IFN-γ and IgG2a that confirm the Th1 responses ^22^. Recombinant plasmids with GRA 5 and MIC3 of *T. gondii* as a cocktail DNA vaccine with low concentration (25 *μg/ml*) could induce partial protection in BALB/c mice ^23^. *GRA7* like other dense granule antigens of *T. gondii* participates in parasitophorous vacuole membrane formation and has a crucial role in establishment in host cells. *ROP2* antigen could express in all *T. gondii* stages of life cycle. Specific clone of human T cells could recognize the *ROP2* and stimulate T cells for producing high levels of IFN-γ ^24^.

## Conclusion

As *GRA7* and *ROP2* could express in two important stages of Toxoplasma life cycle, we prepared this cocktail vaccine. The immunized mice by DNA vaccine produced higher amount of IFNγ, indicated with Th1 response which is confirmed by high level of IgG2a and could increase the survival rate in comparison to control and the other vaccinated groups. These data demonstrate that the cocktail *GRA7*/*ROP2* is a potential vaccine candidate against toxoplasmosis.
